# The Impact of a Phytobiotic Mixture on Broiler Chicken Health and Meat Safety

**DOI:** 10.3390/ani13132155

**Published:** 2023-06-30

**Authors:** Hubert Iwiński, Karolina A. Chodkowska, Kamil Drabik, Justyna Batkowska, Małgorzata Karwowska, Piotr Kuropka, Adam Szumowski, Antoni Szumny, Henryk Różański

**Affiliations:** 1 AdiFeed Sp. z o.o., Opaczewska, 02-201 Warszawa, Poland; 2 Department of Food Chemistry and Biocatalysis, Wrocław University of Environmental and Life Sciences, C.K. Norwida 25, 50-375 Wrocław, Poland; 3 Krzyżanowski Partners Spółka z o.o., Zakładowa 7, 26-670 Pionki, Poland; 4 Institute of Biological Basis of Animal Production, University of Life Sciences in Lublin, ul. Akademicka 13, 20-950 Lublin, Poland; 5 Department of Meat Technology and Food Quality, University of Life Sciences in Lublin, ul. Skromna 8, 20-704 Lublin, Poland; 6 Department of Biostructure and Animal Physiology, Wrocław University of Environmental and Life Sciences, C.K. Norwida 25, 50-375 Wrocław, Poland; 7 Laboratory of Industrial and Experimental Biology, Institute for Health and Economics, Carpathian State College in Krosno, Rynek 1, 38-400 Krosno, Poland

**Keywords:** food safety, phytobiotics, residues, antibiotics alternative, broiler, hepatotoxicity, meat, meat quality

## Abstract

**Simple Summary:**

Food safety is becoming one of the key criteria for consumer choices. More and more importance is being attached to the use of alternatives to classical antibiotic therapy, not only in terms of increasing drug resistance but also because of the risk of residues of substances dangerous to humans. Phytobiotics have become some of the most popular and, what is very important, effective forms of support for the classical antibiotics and their reduction in livestock animals. Therefore, the aim of the study was to examine the effect of a phytobiotic composition that contained several phytoncides—menthol, *trans*-anethole, methyl salicylate, 1,8-cineole, organic acids, and metal ions (Zn and Mn) on meat quality and safety. The results showed that different doses of this mixture (D1—0.5 mL/L, D2—1 mL/L, D3—2 mL/L), used 4 times during the rearing, do not have negative impacts on bird health or meat quality. No residues of menthol, eucalyptol, methyl salicylate, or anethol were found above the limit of quantification in the investigated samples. Furthermore, no residues of the used product were found in tissues intended for human consumption.

**Abstract:**

The purpose of the study was to assess the effects of different doses of a phytobiotic mixture on selected production parameters and meat quality and to assess the residue of the preparation in tissues and the possible toxic effects in broiler chickens. Broiler chicks aged 160 days, divided into four equal groups, were supplemented with the phytobiotic mixture at different doses, D1—0.5 mL/L, D2—1 mL/L, and D3—2 mL/L, four times during a 42-day trial. There were no statistically significant differences in weight gain per week of life and mortality in the birds. The study also demonstrated that the use of the mixture of phytobiotics had no significant effect on colour, pH, WHC, and natural leakage. However, a beneficial effect of the additive was found in the group treated with a dose of 1 mL/L, where less thermal leakage from the meat was demonstrated. Furthermore, significant differences in the change in thigh muscle tenderness were also observed. In the histopathological analysis of the liver no significant differences were observed. In addition, no residues of the mixture or its metabolites were found in the tissues analysed. In conclusion, the proposed scheme of administration of the phytobiotic additive, regardless of the dose, does not cause pathological changes in organs and does not carry the risk of residues of the product in tissues intended for human consumption.

## 1. Introduction

Phytobiotics are currently popular solutions used in broiler breeding. Scientific studies emphasise their effectiveness as supports to classical antibiotic therapy (one of the factors that allow for No Antibiotics Ever (NAE) flocks) as well as in the form of additives that positively affect production parameters and animal welfare, which can be compared to the action of antibiotic growth promoters (AGPs), which have been banned in many countries [1,2,3,4]. The National Office of Animal Health (NOAH, 2001) described antibiotic growth promoters as any medicines used to “help growing animals digest their food more efficiently, get maximum benefit from it and allow them to develop into strong and healthy individuals”. Moreover, AGPs are mostly administered at low, subtherapeutic doses [5]. With controversies related to usage of AGP and radical steps undertaken by many countries, the requirements of quality certificates resulted in bans on the use of AGP, meaning only a ban on adding antibiotics to feed but also in a ban on the use of antibiotics in prophylaxis or metaphylaxis or administering them in subtherapeutic doses, which is observed especially often in the pre/post-thinning period. This is related not only to the fact of increasing drug resistance, but also to the risk of antibiotic residues in tissues intended for human consumption [6]. In the case of phytobiotics, their safeties for both animals and humans are also often underlined. What seems to be crucial in the use of phytobiotics is the absence of dangerous residues in meat. The problem of residues in tissues in the case of antibiotics is still valid, and several publications indicate that it concerns many countries, types of animal production, and mainly antibiotics, in relation to which we observe a decrease in therapeutic effectiveness [7,8]. Nevertheless, there have been very few scientific studies that analyse in detail the possibility of residues or the side effects caused by active substances themselves, and their metabolites, in animal tissues, or the organs of a living animal. While, in the case of some antibiotics used in both human and animal medicine, numerous side effects are known, including hepatotoxicity and nephrotoxicity, in the case of phytobiotics, there are few such reports. In several studies, where the impacts of mainly bactericidal activity were compared, it was also shown that the pathological changes, in organs such as the liver, were smaller than those caused by antibiotics [9]. Moreover, Chodkowska et al. [10] observed that the composition of red pepper fruit, white mustard seed, soapwort root, calamus rhizome, and thymol did not affect biochemical blood parameters in broiler chickens, indicating that these organs were not damaged. What seems interesting is that in previous studies, it has been observed that phytobiotics may positively affect the function of the broiler kidney and liver [11,12]. Many compounds of natural origin, especially those derived from natural essential oils, show positive effects on both carcass and meat quality parameters. An example of this is 1,8-cineole (eucalyptol), the main component of rosemary essential oil and eucalyptus essential oil, which, when added to broiler chicken feed, increases body weight and weight gains and improves the feed conversion ratio (FCR) [13]. Moreover, it can also affect meat shelf life and sensory quality [14]. Another compound that affects the quality of broiler chicken meat is *trans*-anethole. The phytoncides have been proven to improve not only meat quality but also its amino acid composition and fatty acid profile [15]. The composition of phytobiotics used in the study is unique not only because of its composition and the addition of organic acids and metal ions, thanks to which the effect is stronger [16], but also because of the multidirectional action of its active ingredients, which are often perfectly complemented and also cause the phenomenon of the strengthening effect [17,18,19].

Eucalyptol (1,8 cineole) is the terpenoid compound present in plants from the *Myrtaceae*, *Lamiaceae*, and *Zingiberaceae* families. It is most commonly found in the *Eucalyptus* sp., where it makes up 30% to even over 97% of the essential oil composition. Both the compound itself and the eucalyptus essential oil exhibit a range of properties including anti-inflammatory, analgesic, antimicrobial, antioxidant, sedative, and anticancer properties [20,21]. However, it is most commonly used for respiratory problems or for its anti-inflammatory properties. It is widely used in respiratory diseases such as influenza, rhinosinusitis, pneumonia, bronchitis, and asthma. Its properties include being anti-inflammatory and anti-spasmodic or inhibiting mucus secretion due to the regulation of tumour necrosis factor-α (TNFα) or interleukin-1β (IL-1 β), one of the proinflammatory cytokines. Moreover, it may also inhibit the secretion of other inflammatory cytokines by signalling pathways like nuclear factor kappa-light chain enhancer of activated B cells (NF-κB), p38 mitogen activated protein kinase (MAPK), protein kinase B (Akt), trigger receptors expressed on myeloid cells-1 (TREM-1), and Nod-like receptors 3 (NLRP3) inflammasome [21,22,23,24]. The addition of eucalyptus essential oil in broiler chickens’ diets also has positive effects. It can increase BWG, improve cecal microflora balance and thigh muscle fatty acid profile in broiler chickens, decrease FCR, and improve nutrient digestibility and antioxidant activity [25].

*Trans*-anethole is the main constituent in anise seeds (*Pimpinella anisum* L.), star-anise (*Illicium verum* Hooker f.), and fennel (*Foeniculum vulgare* Mill.) essential oils. Its main properties include being anti-inflammatory, antioxidant, and anticarcinogenic. Its anti-inflammatory activity, similar to that of eucalyptol, is based on the inhibition of NF-κB or MAPK and also suppressing lipid peroxidation, transcription factor AP-1, ROI generation, and c-jun N-terminal kinase [26,27]. Moreover, it may decrease alanine aminotransferase (ALT) and aspartate aminotransferase (AST) levels, as well as IL-1β and TNF-α, and increase the levels of interleukin-10 (IL-10) [28].

Menthol is one of the main constituents of the essential oil derived from *Mentha* sp. It has a number of properties, including being antibacterial, anti-helmintic, anti-inflammatory, and antioxidant [29,30,31,32]. The presence of a large number of phenolic compounds, terpenes, and terpenoids or phenylpropanoids is the direct cause of menthol’s strong antioxidant properties. As a result of their interaction with the cell membrane, they cause a number of cell membrane disorders, including the permeability and leakage of cytoplasmic constituents, disrupting its properties and causing it to be damaged [33].

Methyl salicylate is the compound present in many plant’ essential oils but is found in the highest amount in wintergreen (*Gaultheria* sp.). It has various properties including being anti-inflammatory, analgesic, and antipyretic. Additionally, it inhibits cyclooxygenase (COX) and lipooxygenase LOX [34].

The combination of active substances, including metal ions, phytoncides, fatty acids, organic acids, prebiotics and probiotics, often leads to the amplification of their properties and a synergistic effect. Furthermore, as an example, combinations of natural origin substances, like essential oils, with metal ions have significantly better antibacterial or protozoal properties [29,35,36]. Moreover, combinations of essential oils with other active ingredients like organic acids, saponins, alkaloids, or fatty acids improve production parameters like body weight gain (BWG), feed conversion ratio (FCR), and anti-inflammatory activity [16,37,38]. All the aforementioned ingredients and their properties and interactions have key roles in the production parameters, intestinal homeostasis, and animal health in general.

In the era of increasing drug resistance, both in human and veterinary medicine, it seems crucial to develop effective alternatives. In the case of animals intended for meat production, in addition to the therapeutic effect, the effect of stimulating growth and improving production parameters (equivalent to antibiotic growth promoters) [39,40] is expected, while maintaining a high safety margin in terms of affecting important organs such as the liver or kidneys. It is also important because of the use of certain organs (e.g., liver) in human and animal nutrition. In addition, any residues of substances could negatively affect human and animal health in any way in animal tissues used for food production or animal feed. Many phytobiotics have physical and chemical properties that can significantly affect (both negatively and positively) the qualities of tissues intended for human consumption [41,42,43]. For example, extracts and essential oil from mint, which is rich in menthol, are very good preservative agents and can inhibit the growth of many pathogenic bacteria [44,45,46]. What should be taken into consideration is the strong smell of some phytobiotics and related products, especially these used in animal production for the needs of the food industry, which could in turn affect their sensory attributes [14]. Therefore, an increasing number of phytogenic products used in animal nutrition has been analysed for safety reasons by relevant organisations [47,48].

This may affect on consumer choices, related to quality, for e.g., palatability, colour, and smell [49]. For this reason, the evaluation of the impacts of phytobiotic mixtures used on selected meat quality parameters appears to be one of the most important directions of research related to the search for alternatives to antibiotics in animal husbandry.

For the above reasons, phytobiotics are the subject of many studies analysing the possibility of their wider use in farm animals related to food production.

## 2. Materials and Methods

### 2.1. Phytoncides Mixture

The tested composition contained, as predominant compounds, monoterpenoid, phenylpropanoid, aromatic natural compounds—*trans*-anethole, 1,8-cineole, menthol (from essential oils), methyl salicylate, and two organic acids (acetic acid 99%, valeric 99%), as well as zinc acetate dihydrate and manganese (II) chloride (adiBIOTIC^®^ AdiFeed Sp. z o.o., Warsaw, Poland). All compounds were purchased from Sigma-Aldrich (St. Louis, MO, USA) and Centrum Odczynników Chemicznych (Wrocław, Poland) compliant with the Food Chemicals Codex (FCC) and Food Grade (FG) standards. The purity and percentage composition, according to the supplier’s specification, was at a minimum ≥ 95%. The composition of the volatiles used in preparation is presented in Table S1 (Supplementary Materials). 

All phytoncides were mixed with organic acids in equivalent amounts, heated, and left overnight. The prepared mixture was then mixed with an emulsifier (Polysorbate 80, Sigma-Aldrich) for easier dissolution in water. 

### 2.2. Animals and Experimental Design

The experiment material consisted of 160 1-day-old broiler chickens (Ross 308, Aviagen^®^) divided into 4 equal groups, with 40 birds in each group. All groups of birds were vaccinated (at the hatchery) against Newcastle disease (ND) and infectious bronchitis (IB). Vaccination against Gumboro disease (IBD) was performed on day 16 at the farm (also in all groups). All the birds were fed with a commercial diet (Tasomix Sp. z o.o., Alfa feed program, antibiotics free) based on the following program: starter (Day 0–9), grower 1 (Day 10–14), grower 2 (Day 15–35), finisher (Day 36–42). The coccidiostat salinomycin was used in feed from starter to grower 2 for all groups. In the finisher, no coccidiostat was used for any of the groups. Water (in accordance with the standards required for drinking water) was available to the birds ad libitum. During the entire experiment, no acidifying additives or other additives improving quality (e.g., microbiological) were added to the water.

All the flocks were kept under controlled conditions at a room temperature of 32 °C, which was gradually decreased according to normal management practice, and at an 18/6 h light–dark cycle. Body weight was measured weekly from Day 0 to Day 42. 

The differentiating factor was the use of the phytobiotic mixture at different doses: D1—0.5 mL/L, D2—1 mL/L, and D3—2 mL/L. Doses were chosen based on the previously study and studies on an in vitro model related to antimicrobial activity against the most common bacterial species in poultry [2,50]. The control group (C) comprised birds not supplemented with phytobiotics. None of the groups used antibiotics or other drugs during the entire experiment. The birds were kept in deep litter boxes (2 × 2 m), each with the density regulated by the Council Directive 2007/43/EC (33 kg/m^2^) [51].

### 2.3. Phytobiotic Product Distribution

Aqueous solutions of the phytobiotic were prepared immediately before administration (*v*/*v*) in an amount corresponding to the water requirements of the birds according to the recommendation of the chicks’ producer and directly applicated into bell drinkers. The dose of the preparation was administered 4 times, each time for 24 h in drinking water, during the 42-day rearing period on the 4th, 18th, 33rd, and 38th days of the birds’ lives. In order to ensure that the birds fully utilised the preparation, it was the only source of water on each day of administration until it was fully consumed. Then, it was replaced with water from the watering line. During the rearing, the health and mortalities of the birds were recorded, as well as the birds’ body weights (at 7-day intervals).

### 2.4. Tissue Samples Collection

After 42 days of rearing, 8 chickens from each group were randomly chosen and subjected to slaughter, plucking, and evisceration. 

A simplified dissection analysis of the chilled carcasses was performed. The following elements of the carcasses were separated: pectoral muscles, leg muscles (thigh and drumstick), wings, and trunks. On this basis, the slaughter yield (the ratio of the eviscerated carcass to pre-slaughter weight), the proportion of edible giblets in the alive body weight, and the proportion of basic elements in the carcasses were estimated [52]. During dissection, pectoral muscles, leg muscles, livers, and abdominal fat pad samples were collected for further histopathological examination and the analysis of residues of the product in the tissues.

### 2.5. Meat Analysis

#### 2.5.1. pH Value

During the dissection the pH value of the meat was determined in the pectoral (left) and thigh (left) muscles (15 and 60 min, as well as 24 h, after slaughter) using a CP-251 pH meter with a knife-electrode. 

#### 2.5.2. Meat Colour

The colours of both muscles were measured by using the reflectance method on an X-Rite Series 8200 spherical spectrophotometer with the X—Rite Color Master software, using an aperture with a measuring aperture of 25.4 mm in diameter. The measurement was carried out in the 360–740 nm range. A standard D65 illuminant with a standard colorimetric observer with a field of view (observation angle) of 100 was used as the light source. A white standard showed the following parameters: L* = 95.87, a* = −0.49, and b* = 2.39. The results were expressed in units of the CIE LAB [53] system, for which the discriminants reflected the following:

L*—colour brightness; it generally takes positive values and can take values from 0 for a perfectly black body to 100 for a perfectly white body;

a*—chromaticity in the red–green range; it indicates a red colour if it takes positive values and green if it takes negative values;

b*—chromaticity in the yellow–blue range; it denotes a yellow colour if it takes positive values and blue if it takes negative values.

The evaluation of meat colour was carried out twice, before and after heat treatment, and on its basis, the change in the parameters of meat colour was calculated according to the following formula:

(1) $$\Delta E=\sqrt{{\left(\Delta L\right)}^{2}+{\left(\Delta a\right)}^{2}+{\left(\Delta b\right)}^{2}}$$

The following scale was adopted for this parameter:

0 < Δ*E* < 1—the observer does not notice the difference,

1 < Δ*E* < 2—the difference is noticed only by an experienced observer,

2 < Δ*E* < 3.5—the difference is also noticed by an inexperienced observer,

3.5 < Δ*E* < 5—the observer notices a clear colour difference,

5 < Δ*E*—the observer gets the impression of two different colours [54].

#### 2.5.3. Natural and Thermal Leakage

Samples of 100 g were separated from each muscle (right pectoral muscle, right thigh muscle) to determine natural and thermal leakage. Natural leakage, estimated after storing the samples for 24 h at 4 °C, was expressed as a percentage of the initial weight of the meat sample. Thermal leakage was expressed as a percentage of the weight loss of the meat after heat treatment to a temperature of 70 °C at the central point of the sample. The Grau and Hamm [55] method was used to analyse the water-holding capacity of meat, 0.300 g of homogenised muscle were placed on Whatman’s No. 1 filter paper, the paper was placed between two glass plates, and a pressure of 2 kg was applied for 5 min. The resulting leakage was contoured, preserving the sample outline as well, and then planimetered. The result was expressed as the percentage difference in the area of the leakage to the area of the meat sample.

After heat treatment, shear force was measured using Stable Micro Systems’ TA.XT.plus textrometer (Stable Micro Systems Ltd., Godalming, United Kingdom) and a triangular knife. The measurement was made on meat samples cut in the shapes of cubes, each with a side of 10 mm. The cut was made across the samples. The knife travel speed was set at 2 mm/s.

### 2.6. Histopathological Evaluation

The material was fixed in a 4% formaldehyde solution (pH 7.2–7.4) for 24 h, then washed in tap water, dehydrated in alcohol series, and embedded in paraffin. Sections 7 µm thick were stained with haematoxylin and eosin. The material was analysed with a Nikon Eclipse 80i microscope (Nikon Europe B.V., Amstelveen, The Netherlands). All morphometric data were obtained with the use of the NisElementsAr software. The morphometric analysis was performed in the following way. There were 10 counts for every 5 fields of view from each liver from the group, and for each individual, an average was counted. Then, statistical analysis was performed with the use of the Statistica 8.0 software. The differences were tested for statistical significance with a one-way ANOVA analysis of variance.

### 2.7. GC-MS Analysis

The analysis of volatile composition of phytoncides, presented in this paper were determined by the GC-MS technique according to the protocol [56]. The identification of all volatile constituents for untargeted analysis (full-scan mode) was based on the comparison of the mass spectra of the compound obtained experimentally with the mass spectra available in the NIST20 database. Furthermore, the retention indices (RI) obtained experimentally, calculated using macro [57], were compared with the RI available in the NIST20 database and data from the literature [58]. 

The Shimadzu software GCMS Postrun Analysis (Shimadzu Company, Kyoto, Japan) and the ACD/Spectrus Processor (Advanced Chemistry Development, Inc., Toronto, ON, Canada) were used to process the data. The quantification of identified constituents was performed via calculation based on the amount of added internal standard and expressed as a percentage of the integrated peaks’ area. 

Analysis was performed using the Shimadzu 2020 apparatus (Varian, Walnut Creek, CA, USA) equipped with a Zebron ZB-5 MSI (30 m × 0.25 mm × 0.25 μm) column (Phenomenex, Torrance, CA, USA). The temperature of the GC oven was programmed from 50 °C to 250 °C at a rate of 3.0 °C and kept for 3 min. Scanning was performed from 35 to 550 *m*/*z* in electronic impact (EI) at 70 eV and at an ion source temperature of 250 °C. Samples were injected in the split ratio 1:10 and gas helium was used as the carrier gas at a flow rate of 1.0 mL/min. 

For the analysis of volatile compounds presented in the investigated meat, a selected ion monitoring (SIM) mode was applied. Measurement was performed in head-space vials (20 mL) using 1.10 mm solid phase microextraction (SPME)-arrow fibre coated with divinylbenzene-carboxen-polydimethylsiloxane polymer (Shimadzu, Kyoto, Japan). For meat samples, approximately 2 g of homogenised material were placed in a 20 mL headspace vial. Thereafter, the samples were incubated for 10 min at 45 °C and then extracted for 30 min at the same temperature. After the extraction time, the analytes were desorbed in a GC-MS apparatus injector at 250 °C for 3 min according to [59]. To determine the limit of detection (LOD) as well as the limit of quantification (LOQ), meat spiked with investigated analytes was measured in the abovementioned conditions. Based on spectra obtained in full-scan mode, as well as those presented in NIST20, the following ions were chosen for quantification: 60 (valeric acid), 154 (eucalyptol); 71, 81 (menthol); 120, 152 (methyl salicylate); and finally, 148 for anethol. 

### 2.8. Statistical Analysis

The data obtained were subjected to statistical analysis using the SPSS 24.0 package [60]. The distribution of the data was evaluated using the Kolmogorov–Smirnov test, followed by one-way analysis of variance with Tukey’s multiple comparisons tests. The χ2 test was used to statistically verify the values of non-parametric characteristics.

## 3. Results

### 3.1. Effect of Phytogenic Product

During the experiment, the survival rates of the birds supplemented with the tested preparation and the birds’ weekly weight gains (Table 1) were observed. Although no significant differences were found in the percentages of mortality during the rearing, numerically, the lowest values were found for the highest dose of phytobiotic tested. The survivabilities for the groups were 90%, 87.5%, 95%, and 95% for C, D1, D2, and D3, respectively. There was also no effect of the administration of the preparation, regardless of the dose, on the weight gain per week of life of the birds.

### 3.2. Effect of Phytogenic Combination on Meat Parameters and Quality

#### 3.2.1. Carcass Parameters 

Dissection analysis (Table 2) showed that there were no significant differences in terms of slaughter yield or proportion of carcass cuts, although the birds differed significantly in body weight. Liver weights were highest for the control group and D2. However, abdominal fat pad weight was found in the C and D1 groups. In the cases of gizzards and hearts, the highest weight could be found in birds from group D3.

#### 3.2.2. Meat Colour

The analysis of meat colour (Table 3), as one of the basic determinants of the purchasing behaviours of poultry meat consumers, showed that the application of the phytobiotic preparation did not negatively affect the changes in colour coordinates for either raw or thermally treated meat.

#### 3.2.3. pH Values and Natural and Thermal Leakage

Significant differences between the groups included in the study were found in the case of thermal leakage analysis for both muscles analysed (Table 4). It was found that in the case of the pectoral muscle, the lowest value of this trait was found in the muscles of birds supplemented with phytobiotics at a dose of 1 mL/L. Similar observations also applied to the thigh muscle.

Significant differences were also observed in the changes in thigh muscle tenderness. The thigh muscles of the birds in the control group were characterised by the lowest force required to cut muscle fibres. The values of this feature for the tested groups was highest in the D1 group and lowest for D2. The groups supplemented with higher doses of the preparation (1 and 2 mL/L, respectively) did not differ significantly from the other groups included in the study. 

### 3.3. Histopathology

**Figure 1:**
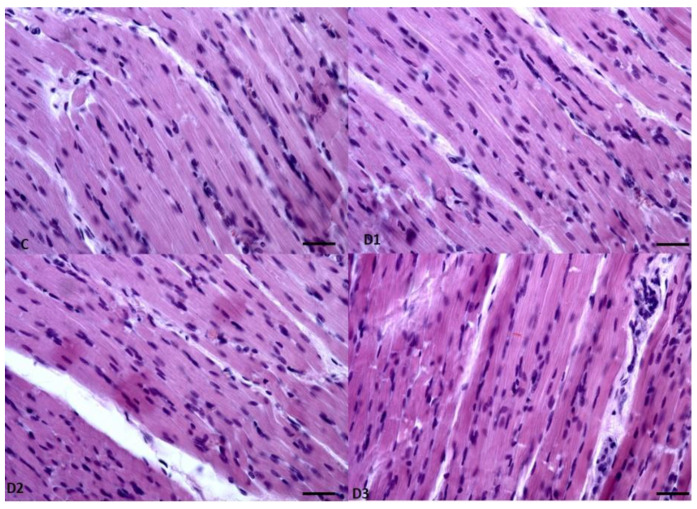
Example pictures of the skeletal muscles from chickens from all the groups. HE, Mag 400×, Scale bar 100 µm.

**Figure 2:**
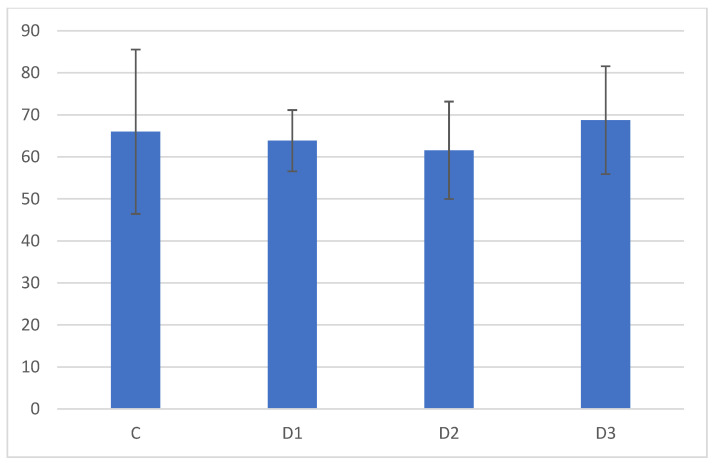
Results of the morphometric analysis of myocyte diameters (µm). All differences are not statistically significant. Note the highest standard deviation in the control group.

**Figure 3:**
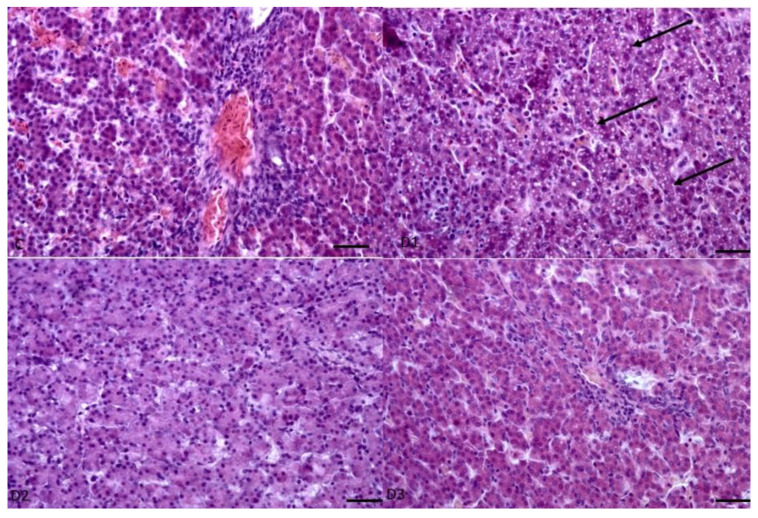
Example pictures of the livers of chickens from all the groups. The presence of large lipid droplets in hepatocytes is marked with arrows. HE, Mag 400× scale bar 100 µm.

**Figure 4:**
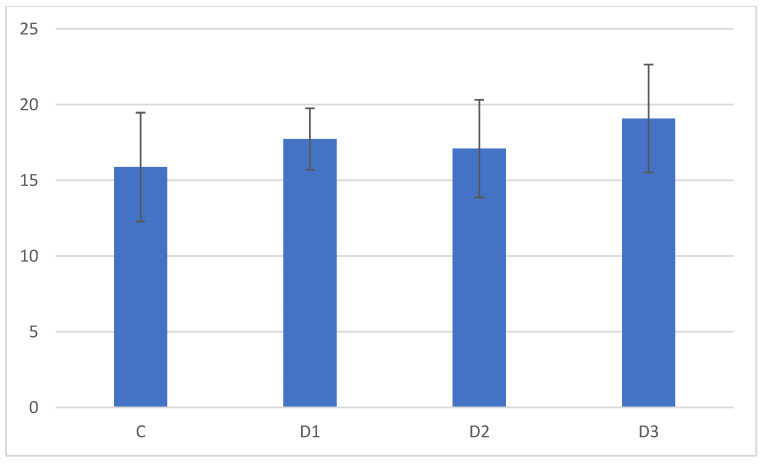
Results of the morphometric analysis of hepatocyte diameters (µm). All differences are not statistically significant. Note the highest standard deviations in the control and D3 groups.

Analysis of the muscles collected from the chickens did not reveal any important differences. The detailed morphometrical analyses did not reveal any statistically significant differences between the groups at this level of the experiment. The muscles were moderately developed, showing typical skeletal muscle structure and size (Figure 1 and Figure 2). Similar to muscles, in the livers also, there were no changes in the tissues and cells (Figure 3 and Figure 4). The morphology of the hepatocytes did not reveal dystrophy or hypertrophic changes in the cells. Interestingly, in all groups except the control group, the hepatocytes contained visible lipid droplets, however this may appear due to the individual features of the chickens (Figure 3). In morphometry, in both counted cells, the control group was characterised by the highest standard deviation, which showed that in this group, the highest variations between individuals were observed; however, for the livers, the D3 group showed similar variation.

### 3.4. Residues in Tissues

Analyses of the phytoncides revealed the presence of compounds from individual essential oil components and volatile components of the product’s ingredients. Table S1 shows the profiles of the main compounds present in the formulation. Pentanoic acid, menthol, eucalyptol, methyl salicylate, and *trans*-anethol were determined as the main compounds in the untargeted full-scan analysis. Therefore, it was decided to optimise the targeted analysis process for the listed substances using the SPME-ARROW technique. The LOQ and LOD levels are shown in Table 5. None of the meat samples showed residues above the instrument detection limits. Only for two samples from groups D3, volatile phytoncides residues were found, but they were below the determination threshold. It should be noted that the LOQ was above the detection level of the substance. This demonstrates the lack of influence of the tested additives on the sensory qualities of the meat.

The composition used in the phytobiotic mixture contains ingredients that are not characteristic of meat products, and so, there was concern about their negative impact on sensory characteristics. This was very important due to the presence of methyl salicylate in the mixture, characterised by a medicinal wintergreen warm-spicy sweet fruity grape rooty-fruity aroma [http://www.thegoodscentscompany.com/data/rw1008472.html, accessed on 23 February 2023]. It is undesirable in foods such as meat. The use of phytoncides that affect the radical change of the aroma characteristic to meat may disqualify it in practical use. 

The technique of using gas chromatography combined with a gas spectrometer did not identify residues of the selected volatile compounds above the limit of quantification shown in Table 5. Only in single samples from groups D3 and D2, eucalyptol methyl salicylate and menthol, were visible at the chromatograph. The results of the carcass samples are presented in Table 6. Their labelled concentrations in the gas phase were below the limit of quantification. This demonstrates, unequivocally, the lack of influence of the preparation used on the sensory characteristics of the products obtained, i.e., the pectoral muscle, liver, and thigh muscles.

## 4. Discussion

The mixture of phytobiotics used in the present study had no significant effect on weekly body weight gain and birds’ mortalities. Furthermore, it can also be observed numerically that the fatness of the birds decreases with an increase level in the phytobiotics used, which may be indicated by a lower proportion of abdominal fat. The lack of statistically significant differences in this respect may be due to the high individual variability of birds. However, it should be noted that, despite the application of relatively high doses of the preparation, it had no negative effect on liver weight. Moreover, the application of this additive did not have a significant statistical effect on the change of meat colour (L*a*b*) in the study groups. A study performed Schlemper et al. [61] and Joseph et al. [62] showed no effect of the addition of the phytobiotic on meat colour. On the other hand, Ashour et al. [63] described that values of L* and a* grads of samples from broiler chicken were lower in a control group than in groups fed with a herbal mixture powder consisted of *Capsicum annuum*, *Thymus vulgaris*, *Salvia Rosmarinus*, *Pimpinella anisum*, *Mentha spicata*, *Nigella sativa*, *Allium sativum*. Other authors noted more yellowish (negative for consumers in some cases) [64] or lighter colour [42,65] which is positively received by consumers [66].

Meat pH is an important factor that occurs during rigor mortis and may affect other meat quality parameters like the texture, colour, and WHC. In this study no significant changes in the pH level (pH15, pH60, pH24) which is consistent with the observations made by, who in his work, in which he also used the phytobiotics mixture, did not show differences in pH, explaining this phenomenon as possibility to prevention the pH from dropping too quickly by the bioactive substances, essential oils and antioxidants contained in phytobiotics which may donate of H + ions contain in antioxidants [67]. Thermal leakage, which has a great impact on the quality properties of meat for culinary purpose was one of the parameters that showed significant differences between the study groups. A beneficial effect of the additive was found in the group treated with dose of 1 mL/L, where less thermal leakage from the meat was demonstrated. Several previous studies showed that strong antioxidant properties of the phytobiotics mixture may prevent the leakage and increase meat quality [1,68].

Histopathological analysis did not reveal any changes in the examined tissues that could suggest a toxic effect of the additive used in the study. An interesting fact is that the lipid droplets were randomly observed in all groups in which the phytobiotic supplement was administered. This type of phenomenon accompanies increased fat metabolism, and in humans and other animals, some disease processes [69,70]. Due to the episodic character of the phenomenon and the inability to perform analyses related to fat metabolism (testing of the profile of fatty acids, cholesterol level in the blood, analysis of metabolic pathways at the level of proteins or genes) and also by the lack of literature describing lipid droplets in conjunction with the use of phytobiotics in poultry.

In the conducted studies, no pathological changes were found, including inflammatory changes, cancer, increased apoptosis or necrosis. Taking into account the obtained results, it can be concluded that in the studied groups no significant differences in the structure of tissues derived from the liver and muscles are observed. The presence of increased lipogenesis in the liver in group D1 is most likely due to processes associated with growth and temporal metabolic changes, especially since fat droplets were present in hepatocytes derived from all groups.

## 5. Conclusions

It can be concluded that the administration of the phytobiotic mixture at different doses, i.e., D1—0.5 mL/L, D2—1 mL/L, and D3—2 mL/L, four times during a 42-day trial did not affect the selected production parameters (like weekly body weight gain and mortality) and several meat quality parameters (colour, pH). What should be particularly emphasised is that the analyses have shown that the preparation does not have a negative effect on the colour of the meat and does not have a damaging effect on organs, regardless of the dose used in the experiment. Furthermore, in none of the cases were the key substances from the phytoncides tested visible, in the GC-MS technique, above the threshold of determination for aroma preservation. Based on the results obtained, as well as the previously conducted in vitro tests for the analysed phytobiotic mixture [2,50], it should be assumed that the preparation may be a safe alternative to classical antibiotic therapy in broiler flocks.

In the era of a growing food crisis related to military operations in the areas of feed and food raw material suppliers, dynamic climate changes, and financial crises in many countries, food safety and maintaining the full supply chain become challenges for many poultry producers. In addition, consumer trends related to microbiological safety and to residues and the reduction of antibiotics in the factory farming of broiler chickens are forcing the entire industry to look for natural, safe alternatives.

This study not only complements the current knowledge about the possibilities of using natural alternatives to antibiotics but also indicates new research directions that should be followed when analysing new phytobiotic products used in animal husbandry, the tissues of which are the raw materials for food production.

## Figures and Tables

**Table 1:** The body weights of the broiler chickens in consecutive weeks of rearing, depending on the doses of preparation.

Age (Days)	Group				SEM
	C Control	D1 0.5 mL/L	D2 1 mL/L	D3 2 mL/L	
0	44.99	43.01	43.44	43.90	0.254
7	215.31	217.35	215.49	207.99	2.743
14	520.28	523.65	522.83	540.47	6.712
21	1058.27	1074.53	1059.07	1066.53	14.129
28	1770.67	1676.67	1608.30	1741.33	26.822
35	2435.12	2480.03	2597.33	2596.10	41.151
42	3109.17	3314.33	3286.67	3251.33	60.820

0.5, 1, and 2 mL/L—doses of phytoncide applied in the experiment.

**Table 2:** The results of the chicken dissections depending on the doses of preparation.

Trait		Group				SEM
		C Control	D1 0.5 mL/L	D2 1 mL/L	D3 2 mL/L	
Body Weight (kg)		2.93 a	3.44 b	3.35 ab	3.22 ab	0.064
Proportions in body weight (%)	Heart	0.371	0.368	0.383	0.398	0.010
	Liver	1.841	1.689	1.749	1.544	0.043
	Gizzard	0.930	0.788	0.878	1.114	0.051
	Abdominal fat pad	0.988	0.929	0.856	0.770	0.053
Carcass yield (%)		80.01	80.04	79.42	80.19	0.210
Proportions in carcass (%)	Breast muscles	35.88	35.89	35.68	35.39	0.334
	Thighs	15.39	15.69	15.00	14.91	0.154
	Drumstics	12.85	12.99	13.19	12.83	0.141
	Wings	9.28	8.80	9.18	9.54	0.121
	Trunk	25.35	25.46	25.86	26.35	0.295

a, b—means in the row differ significantly at *p* ≤ 0.05.

**Table 3:** The results of the chicken meat colour coordinate evaluation, depending on the doses of preparation.

Type of Muscle		Trait	Group				SEM
			C Control	D1 0.5 mL/L	D2 1 mL/L	D3 2 mL/L	
Breast	Raw	L*	55.76	56.05	59.87	55.76	1.041
		a*	−1.24	−1.00	−0.66	−1.13	0.183
		b*	10.34	10.47	10.81	10.45	0.401
		C	10.55	10.67	12.91	10.40	0.459
		H	96.99	95.72	94.43	96.57	0.808
	Cooked	L*	84.73	84.33	85.47	85.50	0.258
		a*	0.54	0.88	0.55	0.32	0.113
		b*	15.25	15.44	15.40	14.97	0.173
		C	15.27	15.47	15.41	14.97	0.174
		H	88.02	87.67	88.05	88.78	0.306
		Δ*E*	29.48	28.83	26.37	30.19	1.077
Thigh	Raw	L*	53.89	56.96	56.85	56.75	0.736
		a*	6.15	3.08	3.64	2.85	0.493
		b*	13.74	13.55	13.66	13.20	0.337
		C	14.28	14.10	14.23	13.13	0.420
		H	73.41	77.67	76.02	82.37	1.476
	Cooked	L*	78.34	78.62	80.36	79.71	0.543
		a*	2.39	2.36	1.88	1.72	0.164
		b*	16.66	16.71	16.47	16.45	0.177
		C	16.87	16.89	16.59	16.57	0.189
		H	81.97	82.04	83.57	83.74	0.503
		Δ*E*	25.11	22.07	23.95	23.45	0.799

**Table 4:** The results of chicken meat technological trait evaluation, depending on the doses of preparation.

Type of Muscle	Trait	Group				SEM
		C Control	D1 0.5 mL/L	D2 1 mL/L	D3 2 mL/L	
Breast	pH15	5.67	5.75	5.63	5.72	0.030
	pH60	5.43	5.40	5.44	5.39	0.023
	pH24	5.46	5.41	5.45	5.40	0.022
	WHC (%)	45.81	45.69	40.11	49.66	1.807
	Natural leakage (%)	1.50	2.26	1.60	1.33	0.156
	Thermal leakage (%)	36.39 c	33.13 bc	27.66 a	28.44 ab	0.894
	Tenderness (N)	49.42	49.46	50.08	50.16	0.207
Thigh	pH15	5.84	5.74	5.79	5.70	0.029
	pH60	5.70	5.63	5.77	5.80	0.033
	pH24	5.76	5.76	5.72	5.72	0.019
	WHC (%)	39.00	34.76	39.31	42.63	1.840
	Natural leakage (%)	1.27	0.78	0.84	1.44	0.120
	Thermal leakage (%)	30.14 b	28.10 ab	23.99 a	24.89 a	0.705
	Tenderness (N)	22.83 a	31.89 b	26.72 ab	28.35 ab	1.150

WHC—water holding capacity; a, b, c —means in the row differ significantly at *p* ≤ 0.05.

**Table 5:** Measured limits of detection and the quantification of selected volatiles.

Compound	*m*/*z*	LOD [ng/g]	LOQ [ng/g]
pentanoic acid	60	0.22	0.65
eucalyptol	154	1.81	5.47
menthol	71	0.46	1.40
methyl salicylate	120	3.96	12.00
*trans*-anethole	148	2.59	7.86

**Table 6:** Results of residues in carcass samples.

	Group			
Carcass	C Control	D1 0.5 mL/L	D2 1 mL/L	D3 2 mL/L
Pectoral muscle (PM)	<LOD	<LOD	<LOD	<LOQ
Thigh muscle (TM)	<LOD	<LOD	<LOD	<LOD
Liver (L)	<LOD	<LOD	<LOD	<LOD
Abdominal fat (AF)	<LOD	<LOD	<LOD	<LOQ

LOD—limit of detection; LOQ—Limit of quantification.

## Data Availability

Data is contained within the article or supplementary materials.

## Supplementary Materials

The following supporting information can be downloaded at: https://www.mdpi.com/article/10.3390/ani13132155/s1, Figure S1: GC-MS (full-scan mode) chromatogram of distilled phytoncides; Figure S2: Sample chromatogram of investigated mixture (SIM and full-scan mode); Table S1: Content (%) of selected phytoncides in analysed mixture.
