# Supplementary material for: The Impact of a Phytobiotic Mixture on Broiler Chicken Health and Meat Safety
Source: Animals (Basel). 2023 Jun 30;13(13):2155. doi: 10.3390/ani13132155 (PMC10339978; doi:10.3390/ani13132155)
Supplement: Supplementary file 1 [file animals-13-02155-s001.zip › animals-2296485-supplementary.pdf]

The Impact of Phytobiotic Mixture on The Broiler Chicken Health and Meat Safety

Hubert Iwiński, Karolina A. Chodkowska, Kamil Drabik, Justyna Batkowska, Małgorzata Karwowska, Piotr Kuropka, Adam Szumowski, Antoni Szumny, Henryk Różański

Crude chromatograms are available under following link:
https://drive.google.com/file/d/1PKc3pal0Wrfq7_aXxTuYa1OkfVyPnjrQ/view?usp=sharing

Table of content

Table S1. Content (%) of selected phytoncides in analysed mixture.. 1

Figure S1. GC-MS (Full scan mode) chromatogram of distilled phytoncides.. 1

Figure S2. Sample chromatogram of investigated mixture (SIM and full scan mode) 2

Table S1. Content (%) of selected phytoncides in analysed mixture

No.	Peak Name	tR (min)	KI exp.	KI NIST	Area (%)
1	Pentanoic acid	5.921	963	904	8.23
2	Eucalyptol	9.568	1033	1032	13.17
3	Menthol	14.352	1175	1175	11.20
4	Methyl salicylate	15.142	1196	1192	44.72
5	*trans*-Anethole	18.151	1286	1286	12.91
Total					90.23

TR – retention time; KI NIST – literature retention index (NIST20); KI exp. - experimental retention index calculated against linear *n*-alkane:

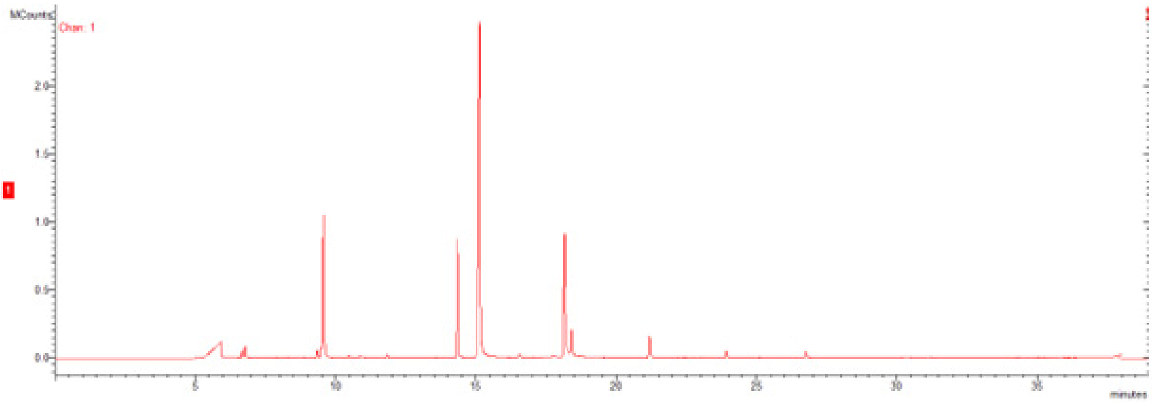

Figure S1. GC-MS (Full scan mode) chromatogram of distilled phytoncides

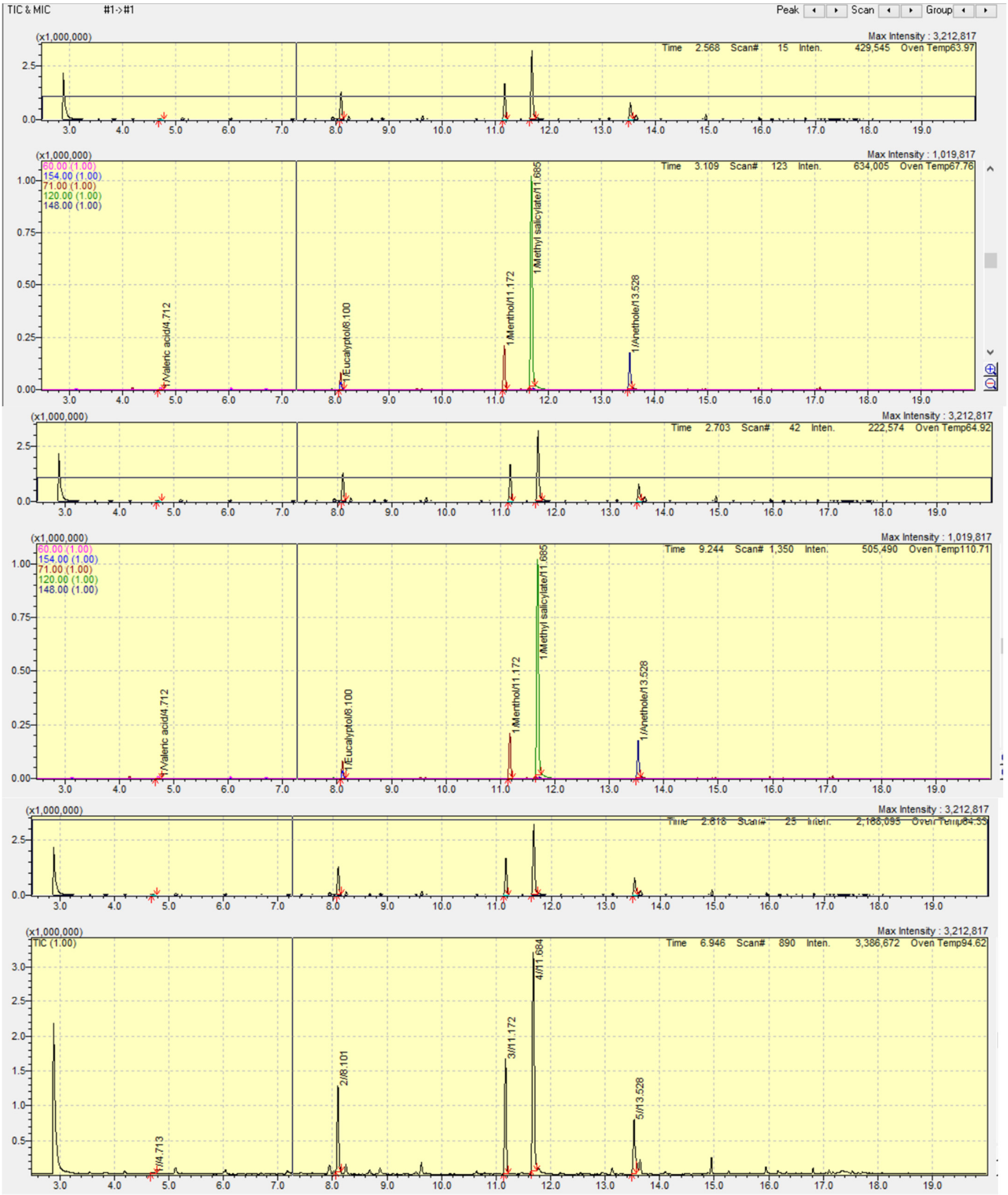

Figure S2. Sample chromatogram of investigated mixture (SIM and full scan mode)
